# T Cell Response in Tuberculosis-Infected Patients Vaccinated against COVID-19

**DOI:** 10.3390/microorganisms11112810

**Published:** 2023-11-19

**Authors:** Luiz Henrique Agra Cavalcante-Silva, Ericka Garcia Leite, Fernanda Silva Almeida, Arthur Gomes de Andrade, Fernando Cézar Comberlang, Cintya Karina Rolim Lucena, Anna Stella Cysneiros Pachá, Bárbara Guimarães Csordas, Tatjana S. L. Keesen

**Affiliations:** 1 Immunology of Infectious Diseases Laboratory, Department of Cellular and Molecular Biology, Federal University of Paraiba, João Pessoa 58051-900, PB, Brazil; luiz0710@gmail.com (L.H.A.C.-S.); erickacg7@hotmail.com (E.G.L.); fernandaalmeida.ufpb@gmail.com (F.S.A.); arthurg.hit@gmail.com (A.G.d.A.); fcezar14@gmail.com (F.C.C.); barbara.guima.csordas@gmail.com (B.G.C.); 2 Infectious and Contagious Disease Complex Dr. Clementino Fraga, João Pessoa 58015-270, PB, Brazil; cintyabeatriz@hotmail.com; 3 Health Secretary of the Paraíba State, João Pessoa 58040-440, PB, Brazil; anna.vigsaude@gmail.com

**Keywords:** infection, immune response, vaccines, lymphocytes

## Abstract

Many studies have focused on SARS-CoV-2 and *Mycobacterium tuberculosis* (*Mtb*) co-infection consequences. However, after a vaccination plan against COVID-19, the cases of severe disease and death are consistently controlled, although cases of asymptomatic and mild COVID-19 still happen together with tuberculosis (TB) cases. Thus, in this context, we sought to compare the T cell response of COVID-19-non-vaccinated and -vaccinated patients with active tuberculosis exposed to SARS-CoV-2 antigens. Flow cytometry was used to analyze activation markers (i.e., CD69 and CD137) and cytokines (IFN-γ, TNFα, IL-17, and IL-10) levels in CD4^+^ and CD8^+^ T cells upon exposure to SARS-CoV-2 peptides. The data obtained showed that CD8^+^ T cells from non-vaccinated TB patients present a high frequency of CD69 and TNF-α after viral challenge compared to vaccinated TB donors. Conversely, CD4^+^ T cells from vaccinated TB patients show a high frequency of IL-10 after spike peptide stimulus compared to non-vaccinated patients. No differences were observed in the other parameters analyzed. The results suggest that this reduced immune balance in coinfected individuals may have consequences for pathogen control, necessitating further research to understand its impact on clinical outcomes after COVID-19 vaccination in those with concurrent SARS-CoV-2 and *Mtb* infections.

## 1. Introduction

In the context of respiratory infections, the COVID-19 pandemic has generated new challenges for patients with pre-existing conditions. The interaction between COVID-19 and tuberculosis (TB) is particularly concerning, a combination that presents a complex clinical landscape [1]. Both respiratory infections exhibit hyperinflammatory patterns characterized by prominent pro-inflammatory cytokines, including TNF-a, IL-6, and IL-1. These released cytokines and chemokines attract immune cells that intensify the pro-inflammatory reactions, thereby contributing to tissue damage [2,3,4]. These findings underscore the gravity of this interaction, revealing that concurrent or sequential pulmonary infections involving COVID-19 and TB lead to exacerbated respiratory symptoms and a pronounced decline in lung function [5,6].

Some studies suggest that tuberculosis may impact the severity of COVID-19 due to compromised immunity and chronic lung inflammation [7,8]. Patients with active tuberculosis exhibit a dysregulated immune response that could impair the immune response to COVID-19, resulting in increased disease severity. Furthermore, COVID-19 can reactivate latent tuberculosis in patients with a history of the disease, further escalating symptom severity [9,10]. It has been demonstrated that *Mycobacterium tuberculosis* (*Mtb*) infection in patients with COVID-19 was more common than other comorbidities such as diabetes, hypertension, and coronary disease. When comparing patients with both TB and COVID-19 to those with pneumonia, 22% of the evaluated TB patients presented with mild/moderate clinical forms of the disease, while the remaining 78% developed more severe forms of COVID [11].

In individuals with latent tuberculosis infection (LTBI), previous contact with and containment of *Mtb* resulted in primed innate immunity. This then triggers the prompt emergence of T cell immunity. This process is accompanied by the formation of cross-reactive heterologous immunity, causing memory T lymphocytes to activate upon exposure to SARS-CoV-2 peptides [12].

An in-depth analysis of transcriptomic patterns drawn from single-cell RNA sequencing in individuals spanning the spectrum of TB infections has revealed heightened signatures linked to COVID-19 risk. These signatures were notably prominent in active TB patients and individuals progressing from latent to active TB [13]. Moreover, monocyte-derived macrophages (MDM) infection with *Mtb* revealed a heightened expression of ACE2 and TMPRSS2. Intriguingly, when MDMs were exposed to conditioned media from *Mtb*-infected MDMs, this exposure not only promoted SARS-CoV-2 infection but also triggered the expression of pro-inflammatory cytokines [13]. These findings align with clinical reports that propose specific responses in TB patients that elevate the susceptibility to severe COVID-19 [14,15]. 

Recent studies indicate a potential connection between SARS-CoV-2 infection and latent tuberculosis (TB) progression to an active state. This could be influenced by factors like host CD4^+^ T cell depletion, lung inflammation, and the activation of stem cell-mediated defenses [16,17]. On the other hand, while the impact of SARS-CoV-2 infection on TB resolution is debated, studies on mice suggest prior *Mtb* infection might protect against SARS-CoV-2-related disease [18]. Also, *Mtb* infection-mediated protection against viruses, such as SARS-CoV-2, has been demonstrated in BCG vaccination and immunity studies, suggesting a potential protection against COVID-19 [19,20,21]. Such findings hint at pathogen interplay, though mechanisms are unclear, underscoring the necessity for further in-depth investigations.

Following the implementation of the COVID-19 vaccination program, there has been consistent control over severe disease and mortality associated with COVID-19. Nevertheless, instances of asymptomatic and mild COVID-19 cases continue to occur in conjunction with cases of TB. Therefore, this study aims to compare the T cell response of COVID-19 non-vaccinated and vaccinated patients with active tuberculosis upon stimulation with SARS-CoV-2 antigens.

## 2. Materials and Methods

### 2.1. Ethics Statement

This study was approved by the National Commission of Ethics in Research (Approval Number: 4.101.879 and certificate CAAE: 31354720.0.0000.5188). All experiments complied with the Declaration of Helsinki’s relevant regulations, institutional guidelines, and ethical standards. Informed consent was obtained from all the enrolled volunteers.

### 2.2. Patient Recruitment 

The recruitment of volunteers took place between May 2020 and July 2023. The study was conducted by 20 volunteers divided into 3 groups: The first group was the control group (CTL, *n* = 9), consisting of healthy volunteers who were not previously vaccinated against COVID-19, were reportedly asymptomatic for the last 10 weeks, were negative via a certified SARS-CoV-2 antibody test (Euroimmun Anti-SARS-CoV-2 assay Perkin Elmer Company, Waltham, MA, USA) and had a negative RT qPCR test for SARS-CoV-2. They were recruited between May 2020 in Brazil, when the original lineage and gamma variants of SARS-CoV-2 were present, and April 2021. The second group was individuals diagnosed with active pulmonary TB caused by *Mycobacterium tuberculosis* and not vaccinated against COVID-19 (NTB group) (*n* = 3). Finally, the third group was the volunteers who had a previous COVID-19 vaccination and were diagnosed with active pulmonary tuberculosis (VTB group) (*n* = 8). TB diagnostics were made through a real-time polymerase chain reaction (PCR) test, known as TRM-TB. Peripheral blood mononuclear cells (PBMCs) were collected in heparinized tubes.

### 2.3. Isolation of PBMCs

Peripheral blood mononuclear cells (PBMCs) collected from the volunteer groups were obtained from heparinized venous blood using density gradient centrifugation (Ficoll-Paque™ Plus, GE Healthcare, Life Sciences, Pittsburgh, PA, USA); the PBMCs were centrifuged for 40 min at 400× *g* and washed three times (200× *g*, 8 min, 4 °C) with phosphate-buffered saline (PBS) (Gibco^TM^, Grand Island, NY, USA) before counting. The cells obtained were resuspended in RPMI 1640 medium, supplemented with 1% antibiotic solution (penicillin, 200 U/mL; streptomycin), 1 mM L-glutamine (1 mM), and 10% fetal bovine serum (Gibco™, Grand Island, NY, USA). Cultures were set up at a concentration of 2.5 × 10^5^ cells/well in 96-well plates in the presence or absence of SARS-CoV-2 antigens (Pool CoV-2, which contained peptides from the spike protein and non-spike proteins, and Pool Spike Cov-2, which had peptides from the spike protein only) and Staphylococcal enterotoxin B from *Staphylococcus aureus* (SEB, Sigma-Aldrich, Saint Louis, MO, USA). The PBMCs were subjected to four different conditions: unstimulated (medium), stimulated with SARS-CoV-2 antigens (Pool Spike Cov-2 and Pool Cov-2, each at 1 μg/well), and stimulated with SEB (1 μg/well). The cells were incubated at 37 °C, 5% CO_2_, for 16 h. Next, brefeldin-A (1 mg/mL, Sigma-Aldrich) was added, and the samples were incubated at 37 °C for 4 h.

### 2.4. Monoclonal Antibodies (mAbs)

The antibodies used for staining were anti-CD4 (APC-Cy7—Clone RPA-T4, cat. 557871), anti-CD8 (APC—Clone RPA-T8, cat. 555369), anti-CD137 (APC—clone 4B4-1, cat. 561702), anti-CD8 (PerCP-Cy5—clone RPA-T8, cat. 555368), anti-CD137 (APC—clone 4B4-1, cat. 561702), anti-CD69 (FITC—clone FN50, cat. 555530), anti-IL10 (APC—clone JES3-19F1, cat. 554707), anti-IL-6 (PE—clone MQ2-6A3, cat. 559331) anti-TNF (PE—clone Mab11, cat. 559321), and anti-IL-17A (PE—clone N49-653, cat. 560486) obtained from BD Biosciences (Franklin Lakes, NJ, USA), along with antibody anti-IFN-γ (PE-Cy5—clone 4S.B3, cat. 25731982) (Invitrogen, Thermo Fisher Scientific, Carlsbad, CA, USA). 

### 2.5. Flow Cytometry Assay

Isolated PBMCs were plated at a concentration of 2.5 × 10^5^ cells per well in a 96-well U-bottom plate. For the extracellular staining, cocktails of mAbs were added and incubated for 30 min at 4 °C. After incubation, the cells were washed with 150 μL of PBS. The plate was centrifuged (8 min, 244× *g*, 4 °C), the supernatant was removed, and 100 μL of 4% formaldehyde and 100 μL of PBS were added to the wells. The plate was incubated at room temperature (25 °C) for 20 min to fix the extracellular staining. After centrifugation (8 min, 244× *g*, 4 °C), the supernatant was removed, and the samples were washed with 150 μL of PBS. The cells were centrifuged again (8 min, 244× *g*, 4 °C) to perform intracellular staining, and the supernatant was discarded. For intracellular staining, the cells were permeabilized with 150 μL of permeabilization buffer (0.5% bovine serum albumin (BSA), *w*/*v* and 0.5% saponin, *w*/*v* in PBS) for 10 min at room temperature (25 °C). After centrifugation (8 min, 244× *g*, 4 °C), the supernatant was removed, and intracellular antibodies were added at a volume suggested by the manufacturer (BD Bioscience, San Jose, CA, USA). Then, the plate was incubated for 30 min at room temperature (25 °C), and after this, 150 μL/well of permeabilization buffer was added. The supernatant was removed after centrifugation (8 min, 577× *g*, 4 °C). Finally, 200 μL/well of Wash B (PBS/BSA) was added, and the samples were transferred to FACS tubes and stored at 4 °C. At least 30,000 gated events were acquired using FACS CANTO II (BD Biosciences, USA) and analyzed using FlowJo v. 10.8 software (BD, Ashland, CA, USA). The BD™ CompBeads Set Anti-Mouse Ig (BD Biosciences, USA) were used as compensation settings during the flow cytometric analyses.

### 2.6. Flow Cytometry Data Analysis

All parameters evaluated on CD4^+^ and CD8^+^ T cells were analyzed using FlowJo software v. 10.8 (BD, Ashland, CA, USA). Limits for the quadrant markers were set based on negative populations (cells) and Fluorescence Minus One (FMO) control. The strategy of the gate is described in Supplementary Figure S1. 

### 2.7. Statistical Analysis

The statistical analyses were performed using GraphPad Prism software version 9. The Shapiro–Wilk test for normality was applied. Two-way ANOVA (followed by Tukey’s post-test) was used for multiple group comparisons. The results were presented as the mean ± standard error of the mean (SEM). The confidence interval was 95%, and values were considered significant when *p* < 0.05. 

## 3. Results

### 3.1. Demographical Characteristics 

We recruited 20 volunteers for this study and distributed them into three groups. The non-vaccinated patients with active TB (NTB) group included three volunteers (three male) with a mean age of 26 (±1.00). Eight volunteers were enrolled in the vaccinated patients with active TB (VTB) group (four males and four females) with a mean age of 34.69 (±3.01). The healthy control was formed of nine donors (five females and four males) with a mean age of 35.63 (±3.05). No significant differences in age were found between the groups. All demographic characteristics are presented in Supplementary Figure S1. 

### 3.2. NTB Patients Express Higher CD69^+^CD4^+^ T Cells than the VTB Group after Viral Antigen Stimulation

**Figure 1:**
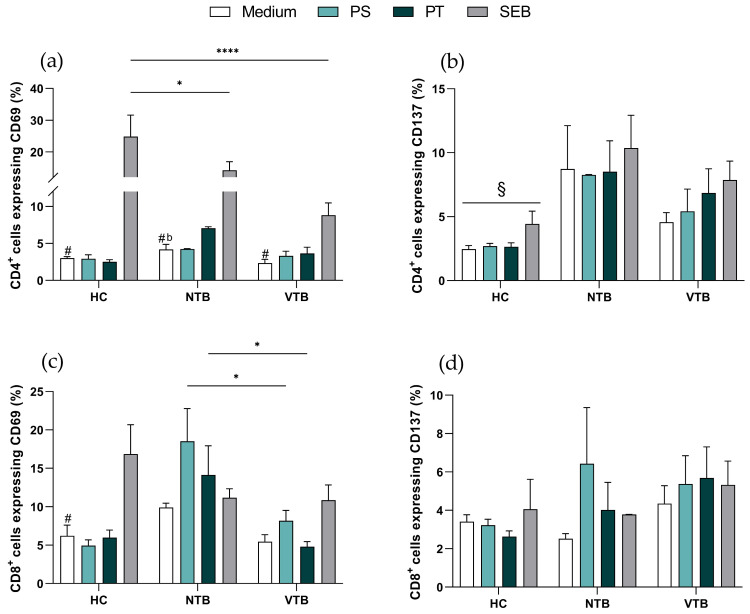
Profile of activation markers in T cells. (**a**) CD69 and (**b**) CD137 expression in CD4^+^ T cells. (**c**) CD69 and (**d**) CD137 expression in CD8^+^ T cells. Graphs are expressed as mean ± standard error (SEM). * *p* < 0.05, **** *p* < 0.0001, ^#^ *p* < 0.05 medium vs. SEB within each group, ^b^ *p* < 0.05 NTB medium vs. HC medium, ^§^ *p* < 0.05 HC vs. NTB; HC = healthy control (*n* = 9); NTB = non-vaccinated with active tuberculosis (*n* = 3); VTB = vaccinated with active tuberculosis (*n* = 8). PS = Pool Spike Cov-2; PT = Pool CoV-2; SEB = staphylococcal enterotoxin B.

Initially, activation marker expression was evaluated in both CD4^+^ and CD8^+^ T cells. In CD4^+^ T cells, there was no difference in CD69 expression between the HC, NTB, and VTB groups when assessed at the base level and after viral stimuli. Conversely, when subjected to SEB stimulation, HC donors exhibited markedly higher levels of CD69^+^CD4^+^ T cells (24.83 ± 6.75) when compared to the NTB (14.20 ± 2.71) and VTB (8.81 ± 1.67) groups. Furthermore, SEB induced greater CD69 expression within all three groups than its corresponding medium condition (Figure 1a). Concerning the evaluation of the CD137 marker, there was no difference between the NTB and VTB groups. However, the HC group consistently exhibited lower levels of CD137^+^CD4^+^ T cells compared to NTB in all conditions (Figure 1b).

Amid CD8^+^ T cells, NTB patients exhibited a notably higher frequency of CD69 expression (PS = 18.53 ± 4.24; PT= 14.13 ± 3.79) compared to patients in the VTB group following viral challenge (PS = 8.17 ± 1.35; PT = 4.79 ± 0.67). In the HC group, SEB stimulation induced a heightened frequency of CD69^+^CD8^+^ T cell (18.84 ± 3.83) compared to unstimulated conditions (Figure 1c). In contrast, no significant differences were observed between the HC, NTB, and VTB groups when CD137 expression was assessed in different conditions (Figure 1d). 

### 3.3. CD8^+^ T Cells from TB Patients Vaccinated against COVID-19 Have Lower Frequencies of TNF-α, While CD4^+^ T Cells Express Higher IL-10 Levels

**Figure 2:**
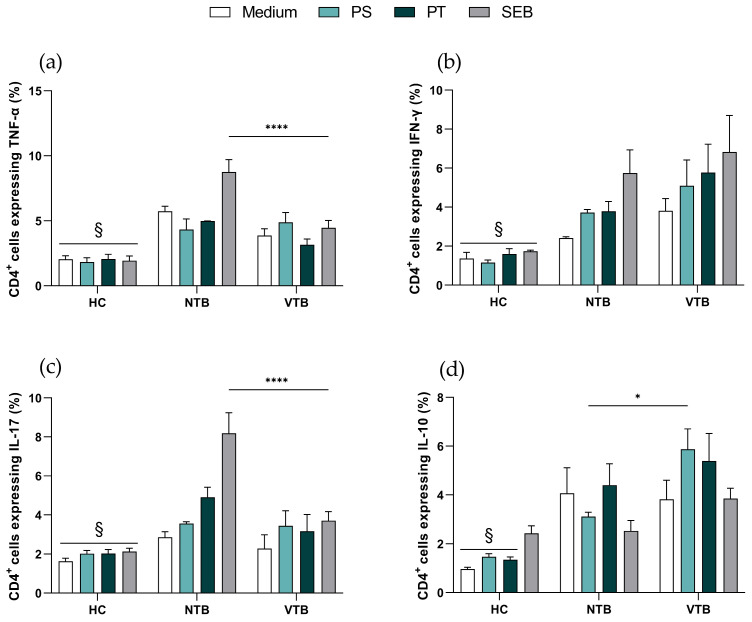
Profile of cytokines in CD4^+^ T cells. (**a**) TNF-α, (**b**) IFN-γ, (**c**) IL-17, and (**d**) IL-10. Graphs are expressed as mean ± standard error (SEM). * *p* < 0.05, **** *p* < 0.0001, ^§^ *p* < 0.05 HC vs. NTB; HC = healthy control (*n* = 9); NTB = non-vaccinated with active tuberculosis (*n* = 3); VTB = vaccinated with active tuberculosis (*n* = 8). PS = Pool Spike Cov-2; PT = Pool CoV-2; SEB = staphylococcal enterotoxin B.

Furthermore, pro- and anti-inflammatory cytokine levels in T cells were assessed. As expected, CD4^+^ T cells from the HC donors exhibited diminished expression levels of pro-inflammatory cytokines, specifically TNF-α, IFN-γ, and IL-17, compared to NTB patients at baseline and following viral antigen stimuli (Figure 2a–c). On the other hand, the frequency of IL-10^+^CD4^+^ T cells was higher in the VTB group (5.88 ± 0.83) compared to the NTB group (3.12 ± 0.17) following the PS stimulus (Figure 2d). 

**Figure 3:**
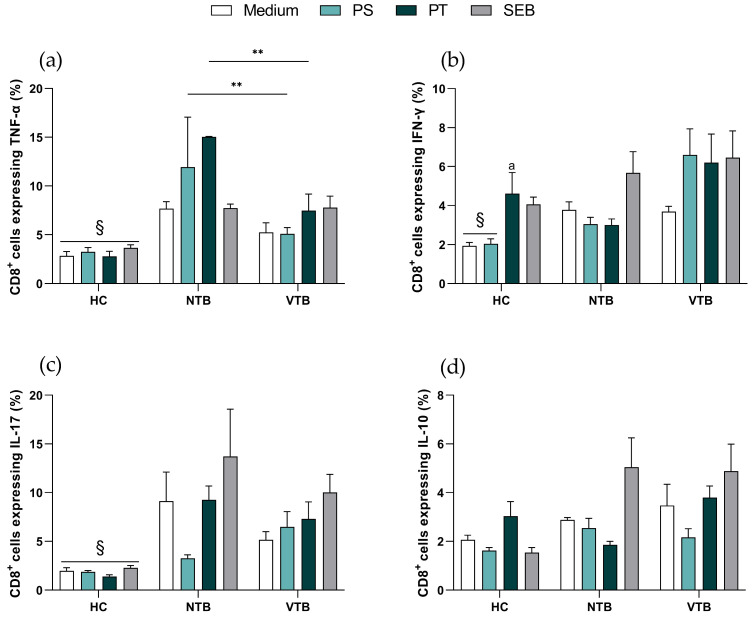
Profile of cytokines in CD8^+^ T cells. (**a**) TNF-α, (**b**) IFN-γ, (**c**) IL-17, (**d**) IL-10. Graphs are expressed as mean ± standard error (SEM). ** *p* < 0.01, ^a^ *p* < 0.05 HC PT vs. HC medium, ^§^ *p* < 0.05 HC vs. NTB; HC = healthy control (*n* = 9); NTB = non-vaccinated with active tuberculosis (*n* = 3); VTB = vaccinated with active tuberculosis (*n* = 8). PS = Pool Spike Cov-2; PT = Pool CoV-2; SEB = staphylococcal enterotoxin B.

In the context of the CD8^+^ T cell findings, it is noteworthy that NTB patients displayed an elevated frequency of TNF-α (PS = 11.94 ± 5.11; PT = 15.03 ± 0.03) in response to viral stimuli, as opposed to the VTB group (PS = 5.09 ± 0.64; PT = 7.47 ± 1.71) (Figure 3a). Similar to what was observed in CD4^+^ T cells, the HC group demonstrated reduced frequencies of IFN-γ and IL-17 expression in CD8^+^ T cells when compared to NTB donors (Figure 3b,c). However, no significant differences were observed across all groups regarding IL-10 expression (Figure 3d).

**Figure 4:**
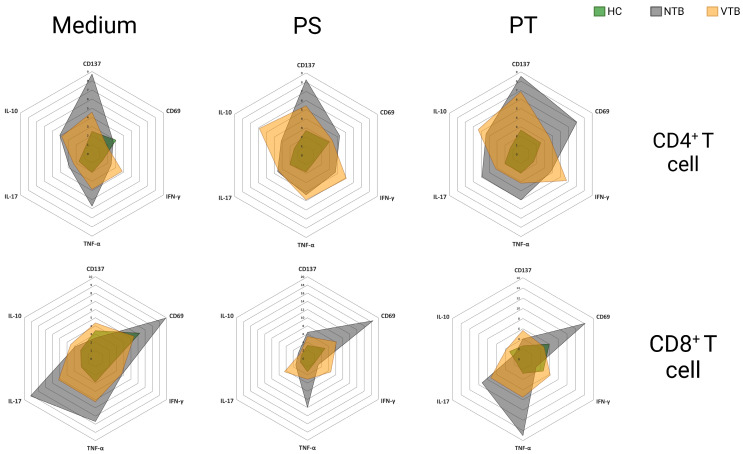
Radar charts of immune profile of CD4^+^ and CD8^+^ T cells. HC = healthy control (*n* = 9); NTB = non-vaccinated with active tuberculosis (*n* = 3); VTB = vaccinated with active tuberculosis (*n* = 8). PS = Pool Spike Cov-2; PT = Pool CoV-2.

The immune profiles of healthy donors and non-vaccinated and vaccinated against COVID-19 patients with active tuberculosis are shown in Figure 4. In addition, all the means ± standard error (SEM) of all parameters analyzed are expressed in Supplementary Tables S2 and S3.

## 4. Discussion

The relationship between COVID-19 and TB has been extensively studied, focusing on co-infection and the effects of SARS-CoV-2 infection on latent TB. This study investigated how COVID-19 vaccination influences TB progression when exposed to viral antigens. Our results indicate a potential decrease in the effector response and an enhancement of regulatory mechanisms upon re-exposure to SARS-CoV-2 peptides.

T cells undergo dynamic alterations in functional and phenotypic characteristics following antigenic stimulation. Notably, they exhibit increased surface expression of activation biomarkers and cytokine production [22,23]. In TB infection, CD69 and CD137 markers were employed to elucidate the state of T cell activation [23,24,25,26]. As described in our findings, CD4^+^ T cells of NTB patients exhibited activation levels comparable to those in individuals of the VTB group. This similarity was evident in the CD69 and CD137 expression levels at baseline and following viral stimulation. These observations suggest that prior COVID-19 vaccination does not significantly influence the activation pattern of CD4^+^ T cells in tuberculosis patients upon re-exposure to the SARS-CoV-2 virus. Furthermore, the CD8^+^ T cells of VTB patients exhibited diminished CD69 expression levels after the viral antigen challenge compared to the NTB patients. 

These results potentially indicate a protective effect for these TB patients in the event of a subsequent SARS-CoV-2 infection. This protective mechanism may arise from avoiding excessive T cell activation, as excessively elevated levels of T cell activation have been linked with unfavorable clinical outcomes [27,28]. Previous studies showed that TB-COVID-19 co-infection reduces the ability to respond to SARS-CoV-2 in vitro [14,29]. Since vaccination against COVID-19 elicits a memory T cell response, the new encounter with SARS-CoV-2 could be more controlled with diminished effects on TB progression.

CD137 is a compelling biomolecular indicator of antigen-activated T cells [23]. Notably, in contrast to the observed outcomes with the CD69 marker, no discernible differences were observed in the CD137 marker expression within the CD4^+^ and CD8^+^ T cell populations. CD69 operates as an early-phase biomarker, manifesting promptly after T cell activation. However, it has an immunoregulatory complex role [30], whereas CD137, a TNFR superfamily member, assumes the role of a co-stimulatory receptor (4-1BB or TNFRS9), attaining functional activity during subsequent phases of T cell activation [31]. In our experimental conditions, the absence of a diminishment in CD137 levels within T cells following viral challenge holds particular significance. This observation is noteworthy as this molecule’s expression is associated with defense against tuberculosis through the modulation of IFN-γ and TNF-α [32].

As CD137 and CD69 markers are closely associated with cytokine production [22,30], the expression of different cytokines in T cells was subsequently evaluated. The interferon response is fundamental to restrain and abrogate viral and bacterial infection [33,34,35], especially IFN-γ, a cytokine associated with the Th1 immune response [36]. The data obtained in this study indicate that a new exposure to SARS-CoV-2 antigens does not modulate the IFN-γ levels in COVID-19-vaccinated patients with active tuberculosis and their non-vaccinated counterparts. It is worth noting that stimulation with the PT antigens within the healthy group increased IFN-γ levels in CD8^+^ T cells compared to their baseline levels. This may have happened due to cross-immunity between SARS-CoV-2 and other coronaviruses, as previously reported [37]. 

When IFN-γ levels are insufficient due to genetic predisposition, immunosuppression, or immune evasion mechanisms employed by the pathogens, the immune system may fail to control COVID-19 and tuberculosis [38]. In COVID-19, reduced IFN-γ levels may contribute to the persistence of viral replication and an increased risk of severe disease, while in tuberculosis, it may result in uncontrolled bacterial growth, dissemination, and the development of active disease [9]. Therefore, the observed persistence of IFN-γ in T cells in our data appears to be a favorable response to new exposure to SARS-CoV-2 antigens in TB patients.

As with IFN-γ, TNF-α is known to be a critical component of the immune response against both tuberculosis and COVID-19. However, elevated TNF-α levels have been linked with immunopathology in severe COVID-19 cases, while in TB, TNF-α is essential for granuloma formation and the containment of the infection [13,15]. Patients diagnosed with pulmonary active tuberculosis develop diverse granulomatous lesions, reflecting a robust inflammatory process primarily driven by cell-mediated immunity [39]. This immunity involves the production of crucial cytokines such as TNF-α, IFN-γ, and IL-12 to combat the *Mtb* infection [40]. When coupled with a coexisting COVID-19 infection, this setting can inflict additional harm to the pulmonary tissue [41]. 

The reduction in TNF-α levels in CD8^+^ T cells following viral stimuli in COVID-19-vaccinated patients with active tuberculosis suggests a potential negative immunomodulatory effect of the combined immune response of vaccination and against *Mtb*. This effect may have protective implications by preventing excessive inflammation and immunopathology during these concurrent infections. Conversely, no significant alteration in IL-17 levels in active TB was observed in CD4^+^ and CD8^+^ T cells. Tuberculosis is associated with a decline in Th17 cell level [42], while COVID-19 immunopathogenesis seems related to elevated levels of IL-17A [43]. The maintenance of IL-17 levels at similar levels in both groups suggests that the impact of the vaccine in active TB patients may not extend to altering the proinflammatory response driven by IL-17. 

To ensure an effective immune response, different regulatory mechanisms must be performed. This phenomenon may happen through several mechanisms, including inhibitory co-stimuli (e.g., CTLA-4) and soluble mediators (e.g., TGF-β and IL-10) [44]. The increase in IL-10 levels in CD4^+^ T cells following spike peptide stimuli in VTB patients may correlate with the reduction in TNF-α. Besides immunoregulatory activity, IL-10 can also trigger antiviral immunity [45]. This antiviral effect of IL-10 is partially associated with its ability to enhance NK cell activity, modifying surface receptors, cytotoxicity [46], and cytokine production [47].

A prior investigation conducted by Andrade and his colleagues [48] revealed that the spike peptides employed in our study induce the activation of CD4^+^ and CD8^+^ T cells, as evidenced by an increased frequency of CD69 expression. Furthermore, it was observed that CD8^+^ T cells exhibit heightened responsiveness to spike-related stimuli when compared to CD4^+^ T cells. The authors also presented evidence of a robust T cell response directed against peptides derived from ORF proteins and the nucleocapsid protein. These observations suggest that healthy individuals previously vaccinated against COVID-19, who are subsequently re-exposed to SARS-CoV-2 antigens, manifest both antiviral and regulatory immune responses.

Rosas Mejia et al. demonstrated that TB-infected mice exposed to a secondary infection with SARS-CoV-2 are resistant to the pathological consequences of secondary viral infection [18]. The authors claim that tuberculosis-infected lungs already host a variety of innate immune cell types that limit the replication of both SARS-CoV-2 or *Mtb* infection and can trigger an adaptive immune response that recognizes SARS-CoV-2 antigens, providing a form of cross-reactive immunity [18]. If we extrapolate these data from mice to humans, we can draw a parallel to what was observed in this study. Vaccination against COVID-19, before *Mtb* infection, generates a cellular memory response that can contain the re-exposure to SARS-CoV-2. Furthermore, as mentioned by the authors, the immune response against *Mtb* itself can reduce the damage caused by COVID-19.

In summary, the reduced presence of CD69^+^CD8^+^ T cells and TNF^+^CD8^+^ T cells and elevated IL-10^+^CD4^+^ T cells in coinfected individuals may reduce inflammation and immunopathological responses. However, this fact prompts inquiries regarding its potential effects on pathogen containment and elimination. To better understand these dynamics, further research is needed to elucidate the mechanisms underlying this phenomenon and to assess its consequences on clinical outcomes in individuals with concurrent SARS-CoV-2 and *Mtb* infections after COVID-19 vaccination.

Our study possesses certain limitations, notably the relatively small sample size within the non-vaccinated group afflicted with active tuberculosis. Additionally, some patients could not recall the specific type of vaccine they received. However, notwithstanding these constraints, our research yields valuable insights into the interplay between COVID-19 and tuberculosis, elucidating the potential impact of vaccination on immune responses in cases of co-infection.

## Data Availability

Data are available on request from the authors.

## Supplementary Materials

The following supporting information can be downloaded at: https://www.mdpi.com/article/10.3390/microorganisms11112810/s1, Figure S1: Flow cytometry data analysis. It was first selected the total lymphocyte gate through SSC and FSC-A profiles, followed by singlet separation using the FSC-A × FSC-H parameters. Next, it was set the CD4^+^ or CD8^+^ T cells using FSC-A x CD8/CD4 parameters. Finally, it was set the markers/CD4 (e.g., IL-17/CD4) or CD8 (e.g., TNF-α/CD8); Table S1: Gender, age, and ethnic-racial self-classification, comorbidities of donors; Table S2: Means ± standard error (SEM) of all parameters analyzed in CD4^+^ T cells.; Table S3: Means ± standard error (SEM) of all parameters analyzed in CD8^+^ T cells.
