# Supplementary material for: T Cell Response in Tuberculosis-Infected Patients Vaccinated against COVID-19
Source: Microorganisms. 2023 Nov 19;11(11):2810. doi: 10.3390/microorganisms11112810 (PMC10673403; doi:10.3390/microorganisms11112810)
Supplement: Supplementary file 1 [file microorganisms-11-02810-s001.zip › microorganisms-2662335-supplementary.pdf]

Supplementary Data

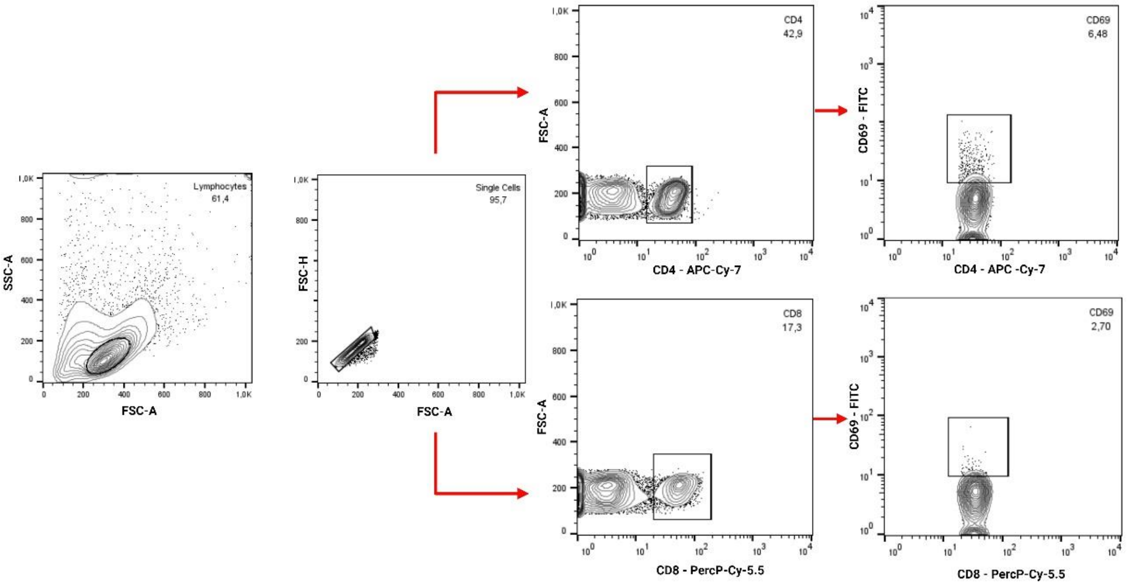

Supplementary Figure S1. Flow cytometry data analysis. The total lymphocyte gate was first selected through SSC and FSC-A profiles, followed by singlet separation using the FSC-A × FSC-H parameters. Next, the CD4⁺ or CD8⁺ T cells were set using FSC-A x CD8/CD4 parameters. Finally, the markers/CD4 (e.g., CD69/CD4) or CD8 (e.g., CD69/CD8) were set.

Supplementary Table S1. Gender, age, and ethnic-racial self-classification, comorbidities of donors.

	**HC**	**NTB patients**	**VTB patients**
**Volunteers**	9	3	8
**Genger – F⁺/M⁺**	5F/4M	3M	4F/4M
**Age (S.E.M.#)**	35.63(±3.05)	26 (±1)	34.69 (±3.01)
		**Ethnic-racial self-classification**	
**Brown**	44.4%	66.67%	50%
**Black**	x	x	25%
**White**	44.4%	33.33%	25%
**Indigenous**	11.1%	x	x
		**Comorbidities**	
**Diabetes (%)**	x	x	50%
**Insuficiência cardíaca (%)**	x	x	12.5%
**Hypertension (%)**	x	x	12.5%
**Asthma (%)**	x	x	25%
**Others (%)**	x	x	12.5%

F, Female; M, Male. HC = health control; NTB = non-vaccinated with active tuberculosis; VTB = vaccinated with active tuberculosis.

Supplementary Table S2. Means ± standard error (SEM) of all parameters analyzed in $CD4^+$ T cells.

**CD137**	**HC**	**NTB**	**VTB**
Medium	2.45 ± 4.56	8.73 ± 3.38	4.56 ± 0.75
Pool Spike CoV-2	2.69 ± 5.41	8.26 ± 0.03	5.41 ± 1.74
Pool CoV-2	2.64 ± 6.85	8.52 ± 2.41	6.85 ± 1.89
SEB	4.42 ± 7.87	10.37 ± 2.55	7.87 ± 1.47
**CD69**	**HC**	**NTB**	**VTB**
Medium	3.00 ± 0.20	2.69 ± 0.33	1.85 ± 0.41
Pool Spike CoV-2	2.92 ± 0.55	2.25 ± 0.52	3.31 ± 0.63
Pool CoV-2	2.51 ± 0.29	7.06 ± 0.18	3.63 ± 0.83
SEB	24.83 ± 6.75	14.20 ± 2.71	8.80 ± 1.67
**TNF-α**	**HC**	**NTB**	**VTB**
Medium	2.04 ± 0.26	5.73 ± 0.39	3.87 ± 0.56
Pool Spike CoV-2	1.82 ± 0.33	4.33 ± 0.81	4.88 ± 0.75
Pool CoV-2	2.05 ± 0.36	4.98 ± 0.003	3.15 ± 0.44
SEB	1.93 ± 0.35	8.75 ± 0.95	4.46 ± 0.56
**IFN-γ**	**HC**	**NTB**	**VTB**
Medium	1.36 ± 0.31	2.41 ± 0.06	3.81 ± 0.62
Pool Spike CoV-2	1.15 ± 0.14	3.71 ± 0.16	5.09 ± 1.32
Pool CoV-2	1.59 ± 0.27	3.79 ± 0.49	5.77 ± 1.45
SEB	1.73 ± 0.06	5.74 ± 1.19	6.82 ± 1.87
**IL-17**	**HC**	**NTB**	**VTB**
Medium	1.63 ± 0.15	2.86 ± 0.28	2.27 ± 0.70
Pool Spike CoV-2	2.02 ± 0.16	3.56 ± 0.09	3.44 ± 0.76
Pool CoV-2	2.02 ± 0.20	4.90 ± 0.51	3.17 ± 0.84
SEB	2.12 ± 0.17	8.19 ± 1.04	3.71 ± 0.45
**IL-10**	**HC**	**NTB**	**VTB**
Medium	0.96 ± 0.07	4.07 ± 1.04	3.81 ± 0.78
Pool Spike CoV-2	1.47 ± 0.12	3.12 ± 0.17	5.87 ± 0.83
Pool CoV-2	1.35 ± 0.11	4.40 ± 0.87	5.39 ± 1.13
SEB	2.43 ± 0.30	2.53 ± 0.42	3.85 ± 0.42

HC = health control (n = 9); NTB = non-vaccinated with active tuberculosis (n = 3); VTB = vaccinated with active tuberculosis (n = 8). PS = Pool Spike Cov-2; PT = Pool CoV-2; SEB = staphylococcal enterotoxin B.

Supplementary Table S3. Means ± standard error (SEM) of all parameters analyzed in CD8$^+$ T cells.

**CD137**	**HC**	**NTB**	**VTB**
Medium	2.41 ± 0.35	2.52 ± 0.93	4.35 ± 0.93
Pool Spike CoV-2	3.22 ± 0.31	6.42 ± 2.93	5.38 ± 1.46
Pool CoV-2	2.63 ± 0.28	4.02 ± 1.44	5.69 ± 1.44
SEB	4.06 ± 1.55	3.78 ± 0.009	5.32 ± 1.23
**CD69**	**HC**	**NTB**	**VTB**
Medium	6.22 ± 1.38	9.90 ± 0.97	5.44 ± 0.92
Pool Spike CoV-2	4.95 ± 0.74	18.53 ± 4.24	8.17 ± 1.35
Pool CoV-2	5.99 ± 0.96	14.13 ± 3.79	4.79 ± 0.67
SEB	16.83 ± 3.83	11.16 ± 1.17	10.86 ± 1.97
**TNF-α**	**HC**	**NTB**	**VTB**
Medium	2.83 ± 0.46	7.66 ± 0.72	5.24 ± 0.97
Pool Spike CoV-2	3.25 ± 0.44	11.94 ± 5.11	5.08 ± 0.64
Pool CoV-2	2.79 ± 0.52	15.03 ± 0.03	7.47 ± 1.70
SEB	3.56 ± 0.31	7.72 ± 0.41	7.79 ± 1.16
**IFN-γ**	**HC**	**NTB**	**VTB**
Medium	1.93 ± 0.17	3.78 ± 0.40	3.69 ± 0.26
Pool Spike CoV-2	2.04 ± 0.25	3.04 ± 0.34	6.59 ± 1.33
Pool CoV-2	4.61 ± 1.08	3.00 ± 0.31	6.20 ± 1.46
SEB	4.06 ± 0.37	5.67 ± 1.08	6.46 ± 1.36
**IL-17**	**HC**	**NTB**	**VTB**
Medium	1.98 ± 0.30	9.12 ± 2.98	5.16 ± 0.83
Pool Spike CoV-2	1.87 ± 0.13	3.25 ± 0.35	6.48 ± 1.55
Pool CoV-2	1.41 ± 0.17	9.25 ± 1.41	7.28 ± 1.41
SEB	2.28 ± 0.22	13.7 ± 4.84	10.0 ± 1.86
**IL-10**	**HC**	**NTB**	**VTB**
Medium	2.06 ± 0.18	2.87 ± 0.10	3.47 ± 0.86
Pool Spike CoV-2	1.62 ± 0.12	2.54 ± 0.40	2.16 ± 0.35
Pool CoV-2	3.03 ± 0.60	1.85 ± 0.14	3.79 ± 0.47
SEB	1.53 ± 0.20	5.04 ± 1.21	4.88 ± 1.10

HC = health control (n = 9); NTB = non-vaccinated with active tuberculosis (n = 3); VTB = vaccinated with active tuberculosis (n = 8). PS = Pool Spike Cov-2; PT = Pool CoV-2; SEB = staphylococcal enterotoxin B.
